# Medicine and Surgery in Egypt

**Published:** 1851-06

**Authors:** 


### ECLECTIC DEPARTMENT,

### AND SPIRIT OF THE MEDICAL PERIODICAL PRESS.

### *Medicine and Surgery in Egypt.*

From the Editorial Correspondence of the Boston Medical and Surgical Journal.

Contrary to my expectation, when the last communication was made from Cairo, I have passed three months in Egypt, which has given me a full and satisfactory opportunity of seeing nearly all its great antiquities. After completing the survey, from the shore of the Mediterranean, to the ancient Syene, where stood the tower spoken of by the prophet Ezekiel, stepping into and mounting temples till wearied with the labor, I then twice crossed the Desert of Arabia — having been twenty-one days on the back of a camel. The spare hours have been devoted to collecting such medical and surgical notes of the country as could be relied on, and this letter embraces some of them.

As before remarked, there are three leading maladies in Egypt, for which physicians exert all their skill, but with only a temporary success. First, the plague — about the origin and treatment of which no two agree, any more than in England or our own country in regard to the origin or treatment of cholera. Second, ophthalmia, which receives a diversity of treatment. Third, cholera, which goeth where it listeth, and admits of little palliation by medicine.

As to ophthalmia, which I have seen extensively through the whole Nilotic extent of Egypt, to me there is nothing obscure in its origin there. Filth gives rise to it: it always has, and always will do so, till the customs and habits of the Arabs and Jews, the principal sufferers, are radically changed — but this will not probably happen, nor the disease, in all its aggravated and hopeless forms, cease to exist, while water flows from the mountains of the moon, down the inclined plane of the Nile. Once in a while, the men and women of the country wash their faces; but when they do, they touch almost everywhere but their eyes. They are unstomachable objects at all times, and just as much so after rubbing their faces as before. The margins of the eyelids look red and fretted in the incipient forms of the disease, which makes them still more cautious about touching them with water. An impression exists, that the eyes should not be wet, and thus the angles are always in a bad state, especially in children and infants. Swarms of flies are invariably crowding for a place — and when they fly off, the purulent matter with which their feet are laden is transferred to the eyes of others, and thus, it is probable, the disease is extensively propagated. The dragoman in the service of myself and traveling associates to the first cataract of the Nile, at one time had fearful indications of an acute attack of ophthalmia, which was an anticipated affliction to us, since our intercourse with the people, and the success of our researches, depended essentially on his tongue and eyes. He was urged to bathe his eyes frequently in cold water, and to sleep with a pledget over them, kept well saturated with it. To this he objected, bringing up the old story that nobody dare apply water under such and such circumstances; but I insisted, and by the second day the aspect was completely changed, and within a week he perfectly recovered. In a second instance, a man, officially connected with a public functionary in Cairo, discovered indications of approaching ophthalmia, and was urged to the same course of treatment. He, too, had his whims and prejudices to contend with, but the uncomfortable thought of becoming blind, secured the service of the water, and he speedily recovered. When leeches, the usual preliminary course, are applied, I have observed that no reduction of inflammation follows, although the excessive pain and sense of fullness in the ball, and another disagreeable feeling, that of grains of sands under the lid, may subside. The physicians here deplete, sometimes very much; but the evidence of the bad success of their treatment is found in the multitude of blind men, women and children who overrun the whole valley of Egypt.

In the desert of Arabia, I made it a matter of special inquiry, whether ophthalmia was there; but it was ascertained that the Bedouin Arabs, those dreaded nomads, whose hand is against everybody, and everybody's hand against them, are not only never subject to attacks of this form of disease, but are almost invulnerable to every other form. They are thin, spare, tall, bronze-colored people, with coal-black, restless eyes, full of activity and deviltry. My recollections of them are painfully vivid, from the circumstance, that while traveling in company with a large mercantile caravan of sixty camels from Ramlah, bound to Egypt, over the haji road, we were greatly alarmed, one night, by the rumored attacks of the Bedouins. There were two, at the moment, quartered on the hospitality of the traveling Arabs, who are in more fear of these lawless possessors of the forest, than of the courbash of the sultan.

With me it is a settled point, that extreme personal filthiness and neglect is the cause of ophthalmia in Egypt. The sparkling particles of sand in the desert, it is believed, produce no bad effects. Yet the Bedouins rarely wash themselves, for it is difficult to procure water enough to meet the demands of thirst. They, however wipe their eyes and dry-wash their faces. When an Arab child is born, as I was informed by a lady of extensive and thorough knowledge of the habits of the Arabs, as well as the Levantines, (that is, those born in the country from European, Syrian, Jewish, and other stocks,) the infant is not washed till it is a year old! Mothers, as I noticed repeatedly, take no pains to drive the flies away from foraging on their children's eyes. In the bazars, among the Jew brokers, and various other classes, I have often gazed at them with astonishment and pity, on account of the cluster of flies prowling about their sore, ulcerated optics. Cleanliness would unquestionably prevent the disease; and a consistent antiphlogistic treatment, seasonably adopted, would effectually cure it, when once developed; but as neither the one nor the other is pursued, ophthalmia is destined to be perpetuated in Egypt while inhabited by the races that now occupy it.

From Dr. Abbott, of Cairo, of whom mention has previously been made, and Dr. Farquar, of Alexandria, both distinguished English practitioners of medicine and surgery, I have obtained information touching the character of the predominant diseases of Egypt, in addition to that collected by personal observation.

Typhus is an annual visitant, principally in the cities, and sweeps off very many persons. The foreign residents, of which the Italians and English are predominant, are sufferers by it. Dr. Farquar informs me that intermittents are extensively prevalent in Lower Egypt, and vast numbers die of it. Medications, thus far, have not been successful. The overflowing of the Nile leaves a vast plateau of country in a muddy state for months, as was noticed in passing over the ground in which the fellahs or farmers are obliged to labor, ankle deep, to cover the seed they may have sown. The evaporation under a burning sun has a peculiar influence on the atmosphere, which deranges the vital machinery of the human system. Pulmonary consumption, one would naturally suppose, from the singular customs of the lower orders of people, would be the all-prevailing and incurable disease. Yet the cases are not numerous; and what is worthy of special record, comparatively rare in places where it would at first seem to be most prevalent. Mechanics, and laborers of all denominations, sailors, &c., including females, go entirely barefooted during life. They are partly amphibious near the Nile, being always in the water, regardless of cold or crocodiles. They sleep, in the one thin cotton garment worn through the day, out of doors, on the bare earth, or in damp, mud hovels — but they have generally excellent health.

Infantile diseases, which embrace a long catalogue of undefined ails, carry off immense multitudes of children. Small-pox also prevails. Dr. Farquar says, that mothers invariably stuff their infants with whatever food they have for themselves. They as frequently force bits of carrot down their untried throats, as something more emollient; and to that cause he attributes a great amount of infantile mortality. When they lose their children, they console themselves with the hope of having more. It is the never-ceasing ambition of both sexes, in every condition of life, in Egypt, to have a numerous offspring. So strong is this desire, that women, and some of a very tender age, as has previously been mentioned, visit certain touchstones in different parts of the country — generally some ancient portion of a pillar or block with hieroglyphical figures — which have the reputation of making them fertile, by saying certain prayers over them. Dr. Abbott informed me that he one day went into an obscure yard, in which he had stored some mummies, where, to his surprise, he found a large collection of females, who displayed great energy in jumping back and forth over the embalmed carcasses. On inquiring what they were doing with his property, they excused themselves by saying that they had heard that, by stepping twelve times over a mummy, it would make them fruitful! Possibly their faith may have some fecundating influence. I can not explain the anomaly of this universal desire — for anomaly it is, contrasted with the dread of a numerous offspring in other countries. It has always been so in the Orient, from the remotest epoch, and probably will continue a characteristic of the people through all succeeding ages. Fatal child-birth seems hardly known. Physicians are rarely consulted in cases of obstetrics. Syphilis is widely spread, and sometimes formidable in its desolating effects on the tissues. I have noticed many a nose in the streets of Cairo, minus all below the bridge. It is singular that the dancing girls — known from the earliest historical periods as the *almeh* — who are prostitutes by profession, and numerous over all Upper and Lower Egypt, are quite free from it, according to the report of those who should be credited. I have seen very many of these singular females — and the question frequently came up in my mind, who are they? They are not Arabs; nor are they Copts — that is, descendants of the original inhabitants, the pyramid builders. They have the facial contour of the Malays, and they also strongly resemble the gypsies seen ranging over England. No one could answer questions respecting them, beyond saying, "they came in the boats."

The Turks are addicted to that one atrocious vice for which Sodom and Gomorrah were consumed by fire from heaven; it appertains to every great man in Egypt. It is without a parallel in the annals of wickedness; yet it is not recognized as a crime, and if it were, those who are the most infamously guilty are above the reach of the law. I could relate a multitude of facts, from the lips of eminent medical gentlemen, illustrative of the weight and depth of this abomination of abominations; but as Herodotus said, in speaking of certain mysteries taught him by the priests, when he visited Egypt twenty-three centuries ago, I do not feel at liberty to reveal them. A harem of females is bad enough, but a harem of small boys puts humanity to shame.

There are one or two military hospitals in the country, at the head of which is a foreign physician or surgeon, assisted by the native Arab doctors, who have been trained to the profession at the expense of the pashaw. Dr. Farquar is surgeon of one of these institutions, at Alexandria, at a salary of seventy-five dollars a month. The duties no way interfere with his private practice. But the surgery is small, made up principally of accidents. Tumors are rarely seen; amputations are not frequent, but couching is a common operation. Distorted limbs are hardly ever seen. I only saw two cases of club-foot in Egypt. One was a mendicant in Cairo, who invariably run after me on his hands and knees; and the other a little girl, in the desert, in a caravan.

Cholera has been a desolating scourge here. Coming down the Nile, I saw many of the returning pilgrims from Mecca, who came in at Kannah, from the Corsair road, and took boats. They gave a fearful account of its devastations among the congregated thousands at the shrine of the prophet. In crossing the desert, there was one of the haji going to Constantinople, who informed me that twenty thousand of the pilgrims had died within a few weeks before he left. When I first landed at Alexandria, the first day of November, 1850, the cholera was just subsiding. The following statistics of its fatality were procured from Dr. Farquar, being extracted from a table which he is expecting to publish in England, in some of the periodicals. Whole number of deaths at Cairo, from August first, to September fourteenth — of cholera, 1951; of other disease, 2310. Total, 4261. Population assumed to exceed 200,000. Deaths in Alexandria, from August third to November eleventh, 1850, — of cholera, 711; all other diseases, 2320. Total 3031. This falls far below the popular representations in regard to the fatality of cholera at the time of my arrival. It was the common impression, probably, of the ignorant, that all deaths, at the period of its prevalence, was caused by it. Exaggerated accounts produced an alarm that reached London. While at Naples, Rome, and finally at Malta, very frightful accounts were circulated of the sweeping destruction cholera was making at Alexandria; which has now dwindled down to 711 deaths in a population of over 100,-000,— so assumed in the absence of any census.

On a former occasion, some general remarks were transmitted in regard to the Egyptian school of medicine, the glory of which was exhibited during the influential days of Clot Bey, the Mussulmanized French surgeon, whose name and reputation as an expert operator, and on account of his daring experiments in the plague, are matters of professional history. With the death of his patron, that wise and shamefully-slandered old Mahomet Ali, the sun of his greatness immediately set. However, besides a reputation, he got what every adventurer in the service of the viceroy hopes for, a splendid fortune,— and with it made tracks for belle France, where he is resting upon his oars, and quaffing such bottles of champaigne as he never drank in the palace of Schonbra, although his master indulged him with the possession of everything else. He is accused now of squandering the pashaw's money, in unnecessarily ordering the fabrication of all sorts of surgical instruments, without reference to cost, and that were of no utility, beyond constituting a museum. Say, however, what they may of him,— it being fine sport to kick a dead lion,— Clot Bey was a great man in his day. He made the medical school at Cairo, and during his administration it had some character. Latterly, it has had none. The French influence has died out,— the Italian ought to be kicked out. A more beggarly, sycophantic set of unprincipled toadies never existed. At El Arish, the ancient Rhinocolura — where political offenders, after having their noses cut off, were sent for exile — the spot, within the half-mud fortress, still remaining, in which king Baldwin, of crusade memory, died — the last town of Egypt, on the borders of Palestine — is a quarantine station, on the top of a sand swell, which is confided to the charge of one of these cunning renegadoes. He honestly confessed to me that he was not a physician; and, further, that he knew nothing about the practice of medicine. He further said that he should like my advice in a difficult case within the tabooed enclosure! He also wished to know whether I had any medicines with me, as he had none. He was health officer, and had the full control of the establishment. Afterwards, a dirty, bare-legged fellow, in the wake of a caravan in which I was traveling, accidentally finding out that I was a hakeem, asked assistance on account of a bronchial inflammation. He had consulted the grand hakeem at El Arish, who furnished a powder that was to act like magic, but instead of affording relief, augmented the evil about the soft palate. I found it was wood ashes, which he was directed to have blown down the throat, in large pinches, several times a day, and a lad was actually seen blowing it down with a hollow stick.

At the time of writing, some German medical aspirants are apparently coming into favor, at Cairo, with the court. They have got possession of the medical school, at all events; and possibly, by their gentlemanly address, their show of science, and their apparent sympathy for the poor, may reign in turn. Dr. Greisingen, from Kiel; Dr. Reger, formerly of Tubingen; and Dr. Lantner, of Vienna, I have every reason for believing, are truly learned men. These gentlemen lecture on the different branches in French, which is translated, word by word, to the students, in Arabic. These native incipient doctors are gathered together, not of their own accord, probably no one of them ever voluntarily seeking a professional education. The most promising looking chaps are selected, to be converted into physicians and surgeons — being taken when about fourteen years of age, and forthwith put in the way of learning the elements of anatomy and operative surgery, besides being made familiar with other branches of knowledge considered essential to the position they are destined to hold as army and navy medical officers. They are both clothed and fed from the public purse, and some of them, particularly when Mahomet Ali was alive, with whom the institution originated, were sent to Paris, Montpelier, England, &c., to be put in communication with a higher order of minds than were to be found in the medical staff of his school of medicine. In the three different visits I made to Cairo, staying a week on each occasion, the lectures were not going on — consequently I am not personally familiar with the daily routine of instruction. In the course of four or five years, reference unquestionably being had to the age of the pupil — and, it is probable, a due respect being paid also to the public demands for medical servants — they have a certificate of qualification, and leap at once into a commission of army or marine surgeons. These Arab students are invariably thick-skulled fellows, who never make any great progress, and consequently there is no hope of another Albufeda to write upon science or the antiquities of Egypt. A German medical gentleman from Berlin, who is passing the winter at Cairo, on account of impaired health, told me that there were several of the government proteges in Europe now, perfecting themselves for medical practice among their countrymen. Thus far, none of the Arabians have been put in places of high professional responsibility. They are the journeymen, under the guidance of Europeans of more calibre than has yet been exhibited in the ranks of the home-made faculty.

I cannot deny myself the pleasure of adverting to Dr. Mussey's widely promulgated anathemas against the use of tobacco — the habitual use of which is as much objected to by myself as by that staunch apostle of temperance in eating, drinking, and smoking. But with all his zeal, his philanthropy, his bold arguments, and cogent reasoning on this subject, in Egypt he would find stumbling-blocks in the way of his conclusions, that would be worse to manage than a hogshead of the best Kentucky in the market. Men, from childhood, smoke incessantly in Egypt. They smoke everywhere and under all circumstances. There is no cessation — not an hour when a cloud of curling smoke is not ascending. It is the first and prominent civility to hand a pipe — and smoke you must, or suffer under the imputation of being no gentleman; and were that good man of Cincinnati sitting where this sentence is written, he would himself smoke, like every one else here. People live long enough, in all conscience, notwithstanding this everlasting smoking, for they outlive their usefulness — outlive everything but animal wants — live, some of them, till everybody wishes they were dead! I have not been an inattentive observer of the smoking mania in Germany and other parts of continental Europe; on the contrary, a strict inquiry into the moral and constitutional effects of the habit, very judiciously called a vice, was instituted as I traveled from kingdom to kingdom where it prevailed; and I have arrived at the gratifying conclusion that if persons wish to smoke, they may, and I shall not waste my breath in warring against the habit.

Another kind of smoking is practised in Egypt, and probably in Syria, unknown to us in America, viz., that of Indian hemp. Cigars are charged with it, and there are apartments where individuals may go and draw in, through a long pipe-stem, a kind of smoke that exalts a dirty, bare-footed rascal into an imaginary prince. In a few minutes he sees the gates of a Mahomedan paradise, gazes wildly towards the sky, and laughs till all consciousness passes away, and he falls into a lethargy of considerable duration. I suspect it is hemp, and not opium, as generally supposed, with which cigars are drugged, and made the instruments, in the hands of designing men, in London, and other great cities on the continent, for the perpetration of many dreadful crimes.

I have collected many curious and novel facts, illustrative of the dietetic regimen and social habits of Arabs, Jews, Nubians, Abyssinians, slaves and freemen, with whom I have had as much acquaintance as is desirable, in their own countries; but how or when they are to be used, is uncertain. A knowledge of them would sadly unhinge some excellent theories of our regenerators of society. Were they to attempt the introduction of some of their hobbies into these countries, they would be laughed at as fools; and after the blush of mortification at the absurdity of their moonshine propositions had subsided, they would laugh themselves at their own stupidity and narrow minded conceptions of the elements of humanity.

*On the way to Beyroot, Feb.* 4, 1851.

